# Amputation of Redundant Scrotum in the Treatment of Varicocele

**Published:** 1871-08

**Authors:** 


*Amputation of Redundant Scrotum in the treatment of Varicocele.* By M. H. Henry, M. D.

In this pamphlet, a new instrument for the operation above named is recommended and described by the author. The instrument, called Scrota. Forceps, may prove to be of service to the surgeon, but we shall be better prepared to appreciate it after trial.

